# Ketone bodies and incident heart failure: 20-year results from the prospective British Regional Heart Study

**DOI:** 10.1093/eschf/xvag015

**Published:** 2026-01-14

**Authors:** Khalil Saadeh, A Olia Papacosta, Lucy T Lennon, S Goya Wannamethee

**Affiliations:** Department of Primary Care & Population Health, University College London, London, UK; University Hospitals Birmingham NHS Foundation Trust, Birmingham, UK; Department of Cardiovascular Sciences, University of Birmingham, Birmingham, UK; Department of Primary Care & Population Health, University College London, London, UK; Department of Primary Care & Population Health, University College London, London, UK; Department of Primary Care & Population Health, University College London, London, UK

**Keywords:** Ketone bodies, Heart failure, Biomarkers, Cohort studies

## Abstract

**Introduction:**

Ketone bodies (KBs), acetoacetate, and β-hydroxybutyrate, are an important fat-derived alternative energy source for the heart and have been implicated in the pathogenesis of cardiovascular disease. The study aimed to determine the relationship between total KB (acetoacetate + β-hydroxybutyrate) and KB ratio (acetoacetate:β-hydroxybutyrate) with incident heart failure (HF) in older men.

**Methods:**

Three thousand four hundred fifty-nine men without prevalent myocardial infarction or HF from the prospective cohort British Regional Heart Study were included in the analysis. Ketone body levels were measured by nuclear magnetic resonance spectroscopy. Participants were followed up for a median 15.9 years.

**Results:**

Three hundred seventy-five men developed HF. Total KB was not significantly associated with incident HF {age-adjusted standardized hazard ratios [HRs] [95% confidence interval (CI)] 0.94 [0.84–1.04], *P* = .231 for trend}. However, KB ratio was significantly associated with incident HF (age-adjusted standardized HR (95% CI) 1.12 [1.01–1.24], *P* = .023 for trend). Risk tended to increase with increasing levels of KB ratio from 0.35 with risk significantly raised when the KB ratio was above 0.55 (top decile) even after adjustment for traditional cardiovascular risk factors, inflammatory markers, and NT-pro-BNP [HR (95% CI) for KB ratio >0.55 vs <0.18 (bottom quartile) = 1.60 (1.13–2.27), *P* = .008]. The increased risk associated with elevated KB ratio was more evident in the younger men (age <70 years). When examined by levels of NT-pro-BNP, elevated KB ratio was significantly associated with increased HF risk only in the presence of elevated NT-pro-BNP (>83 pg/ml; above the median) [age-adjusted HR = 1.87 (1.25–2.81)]. Weaker associations were seen in those without raised NT-pro-BNP [HR = 1.23 (0.66–2.27)].

**Conclusion:**

Elevated KB ratio is associated with a significantly increased risk of HF and may serve as a biomarker of HF incidence particularly when NT-pro-BNP is also elevated.

## Graphical Abstract

**Graphical Abstract:**
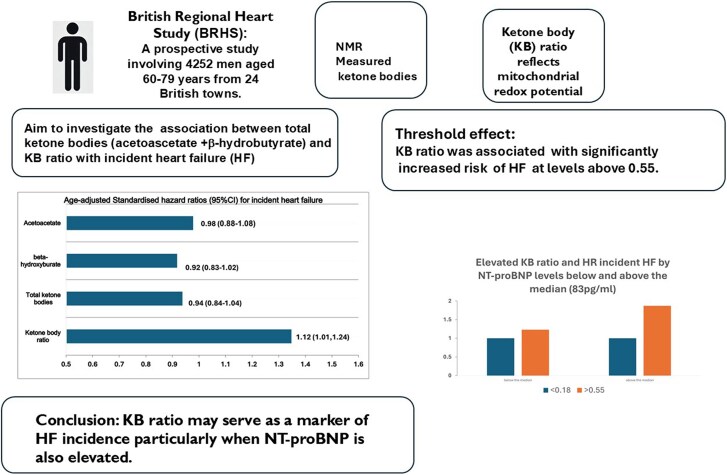

## Introduction

Ketone bodies (KBs) represent an important fat-derived alternative energy source for the heart. Synthesized in the liver, they consist of acetoacetate, β-hydroxybutyrate, and acetone. Their utilization as a source of ATP increases rapidly during physiological stress.^1^ The ratio of acetoacetate to β-hydroxybutyrate, known as the KB ratio, is thought to reflect mitochondrial redox potential [nicotinamide adenine dinucleotide (oxidized/reduced form (NAD^+^/NADH)].^2^ Ketone body also play an important role in several cellular pathways including scavenging free radicals and regulating adrenergic receptor activity.^3^

There is rising interest in the role of KB bodies in cardiovascular disease, particularly heart failure (HF). Studies have demonstrated that the myocardium of patients with HF showed increased use of KB.^4^ Experimental animal and clinical human studies have suggested that KB can influence haemodynamic parameters in the failing heart including improving systolic function.^5–7^ This raised the question of whether KB may act as biomarkers for HF incidence. If so, they may be used to stratify patients at risk of HF, guide prognosis, and suggest therapeutic targets.

Hitherto, the handful of studies that have investigated KB as predictors of HF incidence demonstrate inconsistent findings; some showing positive associations,^8–10^ while others showing no association.^11–13^ In an absence of a review of these studies, major publications have omitted key conflicting findings.

We aim to address these inconsistencies by using a focused population of elderly British male participants [The British Regional Heart Study (BRHS)] to explore the association between total KB, individual KB (β-hydroxybutyrate and acetoacetate), KB ratio (acetoacetate:β-hydroxybutyrate), and incident HF. We also synthesize a review of previous relevant epidemiological studies.

## Methods

### Study population:

The BRHS is a prospective study established in 1978–80, with 7735 male participants aged 40–59 years. These were drawn from 24 towns which were geographically and socioeconomically representative of British men. They were predominantly (>99%) white Europeans. The 20th year follow-up began from 1998 to 2000 and served as the baseline for the current analysis. Data were obtained via a mailed questionnaire (covering lifestyle and medical history), physical examination, and a fasting blood sample. A total of 4252 men (77% of survivors) attended for examination. All men were followed up through general practitioners’ medical records and the National Health Service Register for mortality. Follow-up rate was 99%.^14^

### Follow-up and cardiovascular disease events

Fatal coronary heart disease (CHD) events were defined as death with CHD [*International Classification of Diseases, Ninth Revision* (*ICD-9*) codes 410–414] as the underlying code. Information of nonfatal myocardial infarction (MI) and HF was obtained by regular biennial reviews of participants’ primary care records (including hospital and clinic correspondence) throughout the study period. A nonfatal MI was diagnosed according to World Health Organization criteria. Incident nonfatal HF was based on physician diagnosis of HF recorded in primary care records and verified using available clinical information from primary and secondary care records to ensure consistency with contemporary diagnostic practice^15^; any cases with a strong likelihood of alternative diagnoses were excluded from considerations. Fatal HF cases were those in which the diagnosis of HF was mentioned as the underlying cause of death (*ICD-9* code 428). Incident HF included both incident nonfatal HF and fatal HF cases.

### Cardiovascular risk factors

Details of measurement and classification methods for body mass index (BMI), smoking status, physical activity, social class, alcohol intake, blood pressure (BP), blood lipids and other biochemical markers including N-terminal pro-B-type natriuretic peptide (NT-pro-BNP) and Troponin T, plasma glucose, and renal dysfunction in this cohort have been previously described.^16,17^ Prevalent diabetes mellitus included men with a diagnosis of diabetes mellitus from record review, reported having diabetes on questionnaire, or with a fasting blood glucose level ≥7 mmol/L.

### Measurement of ketone bodies and other metabolites

A high-throughput serum nuclear magnetic resonance (NMR) metabolomics platform was used to quantify >200 metabolite including β-hydroxybutyrate and acetoacetate measured from unthawed serum samples stored at −70°C. Use of this high-throughput metabolomics platform has been widely applied in both epidemiological and genetics studies.^18^ Nuclear magnetic resonance and mass spectrometry–measured values of β-hydroxybutyrate and acetoacetate have been shown to be highly correlated (*R*^2^ = 0.996 and 0.994).^19^ The present analysis uses individual β-hydroxybutyrate and acetoacetate levels, as well as total KB levels (combined β-hydroxybutyrate and acetoacetate) and KB ratio (acetoacetate:β-hydroxybutyrate).

### Exclusion criteria

Men with a diagnosis of HF or previous MI event were excluded from analysis, as were men with missing values of β-hydroxybutyrate and acetoacetate. Analyses were handled using complete-case analysis at that step of the analysis, thus, participants with missing values for any variable within that analytic step were excluded.

### Statistical analysis

Men were divided into equal quartiles of KB levels. Tests for trend for the baseline characteristics across the four groups were performed using the χ^2^ linear test for trend for categorical variables and analysis of variance (ANOVA) for continuous variables. Total KB, β-hydroxybutyrate, and acetoacetate as well as insulin, C-reactive protein (CRP), IL-6, total triglycerides, gamma-glutamyl transferase (GGT), alanine aminotransferase (ALT), and NT-pro-BNP were all-natural log transformed to yield a normal distribution. Kaplan–Meier methods were used to calculate the cumulative incidence of HF by quartiles of total KB and KB ratio. Time of censoring was defined as the last follow-up completed prior to June 2018.

Cox proportional hazards model was used to assess the multivariate-adjusted hazard ratios (HRs) of total KB and KB ratio with incident HF and MI over time, correcting for potential confounders. Total KB, β-hydroxybutyrate, acetoacetate, and the KB ratio were used as quartiles, with bottom quartile used as reference, and as continuous variables whereby Total KB, β-hydroxybutyrate, and acetoacetate were log transformed. As continuous variables, standardized HR were shown to allow interpretation per one standard deviation increase in total, individual, or ratio of KB. Stepwise Cox regression models were used with progressive adjustment: (i) age adjusted, (ii) adjusted for demographic and traditional cardiovascular risk factors: age, BMI, social class, systolic BP, taking BP medication, smoking, physical activity, estimated glomerular filtration rate (eGFR), total cholesterol, HDL, taking lipid-lowering drugs, IL-6 and prevalent atrial fibrillation (AF), and finally (iii) model 2 additionally adjusted for NT-pro-BNP to assess whether the association is independent of established CV risk factors. A *P-*value of <.05 was considered statistically significant. Age-adjusted restricted cubic spline models were used to visually depict a non-linear association between continuous total KB (log transformed) and KB ratio with incident HF with knots at the 5th, 25th, 50th, 75th, and 95th percentiles. All statistical analyses were performed using SAS software, V.9.4 of the SAS System for Windows.

### Supplementary analyses

Three further subgroup analyses were conducted to examine the effects of (i) NT-pro-BNP levels, whereby participants were stratified into levels below and above the median (83 pg/ml); (ii) age, whereby participants were stratified into <70 years and ≥70 years; and (iii) diabetic status, whereby participants were stratified into diabetic and non-diabetic men. We additionally explored whether KB were associated with a particular HF subtype restricting incident cases to those with available echocardiographic-derived information on left ventricular fraction. Thus, participants with known HF subtype were stratified into HF with reduced ejection fraction (HFrEF) and HF with preserved ejection fraction (HFpEF).

## Results

### Study population

A total of 4252 men were examined at the 20th year follow-up conducted from 1998 to 2018. Of those 130 were excluded due to previous HF, 424 were excluded due to previous MI. A further 5 were excluded due to the metabolite value being considered an outlier, and 234 excluded for having at least one of β-hydroxybutyrate or acetoacetate values missing. This leaves a total of 3459 participants included in this analysis. Of these, 375 (10.84%) developed incident HF during a median follow-up time of 15.9 years (6.82 cases per 1000 men per year).

### Baseline characteristics

The average participant had a mean age of 68.5 ± 5.5 years and was overweight (mean BMI 26.8 ± 3.6 kg/m^2^) and hypertensive [mean systolic blood pressure (SBP) 150.0 ± 23.8]. *Table 1* compares the characteristics of men who did, and did not, develop incident HF. Men in the incident HF group had statistically significantly higher age, BMI, systolic BP, hypertension prevalence, NT-pro-BNP, CRP, and IL-6, but significantly lower eGFR.

**Table 1:** Baseline characteristics of men who did, and did not, develop incident heart failure

	Did not develop HF (*n* = 3084)	Developed HF (*n* = 375)	*P*-value
Age (years)	68.36 (5.48)	69.55 (5.34)	**<**.**0001**
BMI (kg/m^2^)	26.72 (3.56)	27.47 (3.67)	.**0001**
Current smoker (%)	408 (13.25)	38 (10.13)	.086
Physical activity (% inactive or only occasionally active)	978 (31.71)	117 (31.2)	.099
Heavy alcohol use (%)	86 (2.83)	13 (3.49)	.474
Social class (% manual occupation)	1567 (50.93)	187 (50)	.931
Systolic BP (mmHg)	149.66 (23.68)	153.04 (24.59)	.**097**
Diastolic BP (mmHg)	85.6 (11.03)	86.04 (10.54)	.463
Hypertension diagnosis (%)	829 (28.16)	131 (36.69)	**<**.**001**
Taking BP-lowering medication (%)	799 (26.28)	138 (37.2)	**<**.**0001**
Diabetes mellitus (%)	331 (10.73)	49 (13.07)	.172
Taking lipid-lowering medication (%)	130 (4.22)	21 (5.6)	.215
Total cholesterol (mmol/L)	6.06 (1.06)	5.99 (1.04)	.244
LDL (mmol/L)	3.92 (0.96)	3.87 (0.94)	.335
HDL (mmol/L)	1.33 (0.34)	1.32 (0.35)	.398
eGFR (ml/min/1.73 m^2^)	73 (12.68)	71.57 (12.41)	.**039**
NT-pro-BNP (pg/ml)	82.27 (41–156)	152.93 (67–320)	**<**.**0001**
Troponin T (pg/ml)	13.3 (24.61)	15.69 (9.15)	.063
CRP (mg/L)	1.65 (0.79–3.31)	1.93 (0.97–3.5)	.**009**
IL-6 (pg/ml)	2.36 (1.53–3.31)	2.64 (1.69–3.64)	.**003**

Values are *n* (%) or mean (standard deviation). For NT-pro-BNP, CRP, and IL-6, values are geometric mean (inter-quartile range).

BMI, body mass index; BP, blood pressure; NT-pro-BNP, N-terminal pro-B-type natriuretic peptide; CRP, C-reactive protein; IL-6, interleukin-6; HF, heart failure. Significant *P*-values are indicated in bold.

### Baseline characteristics by ketone body quartiles

Baseline characteristics and biochemical profile by quartiles of total KB and KB ratio are shown in *Tables 2* and *3*, respectively, and by quartiles of β-hydroxybutyrate and acetoacetate quartiles are shown in Supplementary Tables S1 and S2, respectively. Overall, trends in total KB quartiles were similar to trends in β-hydroxybutyrate and acetoacetate quartiles but more closely matched β-hydroxybutyrate than acetoacetate. This is expected as β-hydroxybutyrate accounts for ∼70% of circulating KB.^1^ However, KB ratio showed different trends to total KB across several demographic variables (e.g. age, BMI, and heavy drinking) and biochemical profiles (e.g. lipid profiles, insulin levels, inflammatory, and hepatic markers).

**Table 2:** Baseline characteristics and biochemical profile by total ketone body quartiles

	Q1 (<0.1786 mmol/L) (*n* = 862)	Q2 (0.1786–0.2481 mmol/L) (*n* = 864)	Q3 (0.2481–0.3820 mmol/L) (*n* = 862)	Q4 (>0.3820 mmol/L) (*n* = 861)	*P*-value
Age (years)	67.59 (5.4)	68.33 (5.44)	68.93 (5.61)	69.13 (5.34)	**<**.**0001**
BMI (kg/m^2^)	26.94 (3.58)	27.11 (3.6)	26.97 (3.78)	26.2 (3.27)	**<**.**0001**
Current smoker (%)	122 (14.19)	109 (12.62)	106 (12.33)	107 (12.44)	.139
Physical activity (% inactive or only occasionally active)	262 (30.39)	293 (33.91)	271 (31.44)	265 (30.78)	.135
Heavy alcohol use (%)	21 (2.46)	11 (1.3)	24 (2.83)	37 (4.36)	.**002**
Social class (% manual occupation)	432 (50.23)	444 (51.39)	443 (51.51)	428 (49.94)	.868
Systolic BP (mmHg)	148.42 (24.3)	148.93 (23.48)	150.98 (23.61)	151.71 (23.56)	.**01**
Diastolic BP (mmHg)	84.84 (10.84)	85.18 (10.86)	85.53 (10.96)	87.01 (11.08)	**<**.**001**
Hypertension diagnosis (%)	249 (30.29)	261 (31.18)	231 (28.66)	216 (26.12)	.113
Taking BP-lowering medication (%)	246 (29.11)	250 (29.24)	226 (26.59)	214 (25.15)	.164
Total cholesterol (mmol/L)	5.92 (1.01)	6.07 (1.09)	6.08 (1.05)	6.12 (1.08)	**<**.**001**
LDL (mmol/L)	3.8 (0.91)	3.92 (0.98)	3.92 (0.95)	4.02 (0.96)	**<**.**0001**
HDL (mmol/L)	1.3 (0.31)	1.27 (0.32)	1.34 (0.35)	1.4 (0.36)	**<**.**0001**
Triglycerides (mmol/L)	1.67 (1.17–2.3)	1.80 (1.27–2.44)	1.63 (1.18–2.18)	1.42 (1.03–1.89)	**<**.**0001**
Taking lipid-lowering medication (%)	38 (4.41)	34 (3.94)	43 (4.99)	36 (4.18)	.740
Diabetes mellitus (%)	151 (17.52)	107 (12.38)	67 (7.77)	55 (6.39)	**<**.**0001**
Insulin (pmol/L)	9.49 (5.96–13.23)	9.03 (5.93–12.8)	8.33 (5.78–12.18)	7.24 (5.01–10.44)	**<**.**0001**
NT-pro-BNP (pg/ml)	78.26 (37–160)	84.77 (42–160)	89.12 (45–164)	103.54 (51–190)	**<**.**0001**
Troponin T (pg/ml)	14.11 (44.49)	13.12 (8.12)	13.36 (8.41)	13.62 (9.24)	.840
CRP (mg/L)	1.49 (0.7–3)	1.73 (0.81–3.37)	1.72 (0.84–3.42)	1.79 (0.82–3.6)	.**003**
IL-6 (pg/ml)	2.16 (1.38–3.06)	2.29 (1.51–3.31)	2.53 (1.64–3.4)	2.61 (1.69–3.81)	**<**.**0001**
eGFR (ml/min/1.73 m^2^)	73.64 (11.84)	72.61 (11.85)	72.62 (14.24)	72.44 (12.5)	.179
GGT (U/L)	28.22 (18–39)	28.79 (20–40)	28.22 (19–38)	28.50 (19–37)	.930
ALT (U/L)	16.28 (13–21)	16.12 (12–21)	15.40 (12–20)	15.18 (12–19)	.**003**
Albumin g/L	44.05 (2.63)	44.15 (2.79)	44.26 (2.63)	44.3 (2.78)	.216
AF (%)	20 (2.33)	31 (3.59)	20 (2.33)	37 (4.3)	.**044**
Incident HF (%)	96 (11.14)	101 (11.69)	84 (9.74)	92 (10.69)	.611
Incident MI (%)	137 (15.89)	124 (14.35)	116 (13.46)	126 (14.63)	.551
Incident stroke (%)	97 (11.25)	108 (12.5)	98 (11.37)	109 (12.66)	.721

For normally distributed continuous variables, values are mean (standard deviation). For insulin, CRP, IL-6, total triglycerides, GGT, ALT, adiponectin, DM, diabetes mellitus, and NT-pro-BNP, values are geometric mean (inter-quartile range). *P*-values for continuous variables were calculated with ANOVA. For categorical variables, values are *N* (% of total) and *P*-values for χ^2^ tests. Significant *P*-values are indicated in bold.

**Table 3:** Baseline characteristics and biochemical profile by ketone body ratio quartiles

	Q1 (<0.3300813 mmol/L) (*n* = 862)	Q2 (0.3300813–0.4010989 mmol/L) (*n* = 862)	Q3 (0.4010989–0.4751381 mmol/L) (*n* = 863)	Q4 (>0.4751381 mmol/L) (*n* = 862)	*P*-value
Age (years)	68.19 (5.51)	68.57 (5.55)	68.73 (5.38)	68.49 (5.46)	.222
BMI (kg/m^2^)	26.77 (3.54)	26.73 (3.62)	26.65 (3.54)	27.07 (3.61)	.075
Current smoker (%)	111 (12.91)	107 (12.41)	119 (13.82)	107 (12.43)	.577
Physical activity (% inactive or only occasionally active)	282 (32.71)	308 (35.73)	260 (30.13)	241 (27.96)	.**007**
Heavy alcohol use (%)	29 (3.42)	17 (2)	24 (2.83)	23 (2.71)	.351
Social class (% manual occupation)	481 (56.06)	426 (49.48)	428 (49.65)	412 (47.91)	.**014**
Systolic BP (mmHg)	151.87 (23.63)	150.88 (24.71)	148.75 (23.34)	148.51 (23.23)	.**006**
Diastolic BP (mmHg)	86.22 (10.61)	85.53 (11.42)	85.48 (10.95)	85.31 (10.87)	.330
Hypertension diagnosis (%)	240 (29.16)	248 (30.24)	227 (27.65)	242 (29.23)	.714
Taking BP-lowering medication (%)	215 (25.35)	230 (27.06)	231 (27.27)	260 (30.37)	.132
Total cholesterol (mmol/L)	6.12 (1.06)	6.03 (1.1)	6.06 (1.02)	5.99 (1.06)	.093
LDL (mmol/L)	4 (0.97)	3.93 (0.97)	3.93 (0.92)	3.8 (0.94)	**<**.**001**
HDL (mmol/L)	1.34 (0.33)	1.35 (0.33)	1.33 (0.34)	1.31 (0.35)	.100
Triglycerides (mmol/L)	1.58 (1.15–2.1)	1.55 (1.13–2.09)	1.62 (1.15–2.21)	1.73 (1.17–2.52)	**<**.**0001**
Taking lipid-lowering medication (%)	21 (2.44)	40 (4.64)	33 (3.82)	57 (6.61)	**<**.**001**
Diabetes mellitus (%)	113 (13.11)	95 (11.02)	73 (8.46)	99 (11.48)	.**020**
Insulin (pmol/L)	8.85 (5.73–12.43)	8.33 (5.55–11.62)	8.33 (5.65–12.1)	8.41 (5.61–11.8)	.103
NT-pro-BNP (pg/ml)	81.45 (41–159)	87.36 (43–161)	90.02 (44–169)	93.69 (45–184)	.095
Troponin T (pg/ml)	13 (7.8)	12.97 (7.06)	14.58 (44.53)	13.65 (10.25)	**<**.**0001**
CRP (mg/L)	1.70 (0.8–3.6)	1.60 (0.74–3.3)	1.65 (0.8–3.3)	1.79 (0.87–3.33)	.224
IL-6 (pg/ml)	2.34 (1.5–3.34)	2.41 (1.58–3.32)	2.36 (1.56–3.28)	2.48 (1.55–3.54)	.213
eGFR (ml/min/1.73 m^2^)	72.34 (12.38)	72.9 (13.83)	72.76 (11.65)	73.31 (12.64)	.461
GGT (U/L)	29.08 (19–40)	27.11 (18–36)	27.94 (19–38)	29.67 (19–40)	.**004**
ALT (U/L)	15.96 (12–21)	15.33 (12–20)	15.33 (12–20)	16.44 (13–21)	.**002**
Albumin g/L	44.32 (2.79)	44.22 (2.84)	44.12 (2.59)	44.11 (2.61)	.343
AF (%)	13 (1.51)	24 (2.8)	31 (3.6)	40 (4.65)	.**002**
Incident HF (%)	84 (9.74)	90 (10.44)	86 (9.97)	113 (13.1)	.090
Incident MI (%)	140 (16.24)	115 (13.34)	124 (14.37)	124 (14.39)	.387
Incident stroke (%)	99 (11.48)	102 (11.83)	117 (13.56)	94 (10.9)	.361

For normally distributed continuous variables, values are mean (standard deviation). For insulin, CRP, IL-6, total triglycerides, GGT, ALT, adiponectin, and NT-pro-BNP, values are geometric mean (inter-quartile range). *P*-values for continuous variables were calculated with ANOVA. For categorical variables, values are *N* (% of total) and *P*-values for χ^2^ tests. Significant *P*-values are indicated in bold.

### Association of plasma ketone bodies with incident heart failure

**Figure 1:**
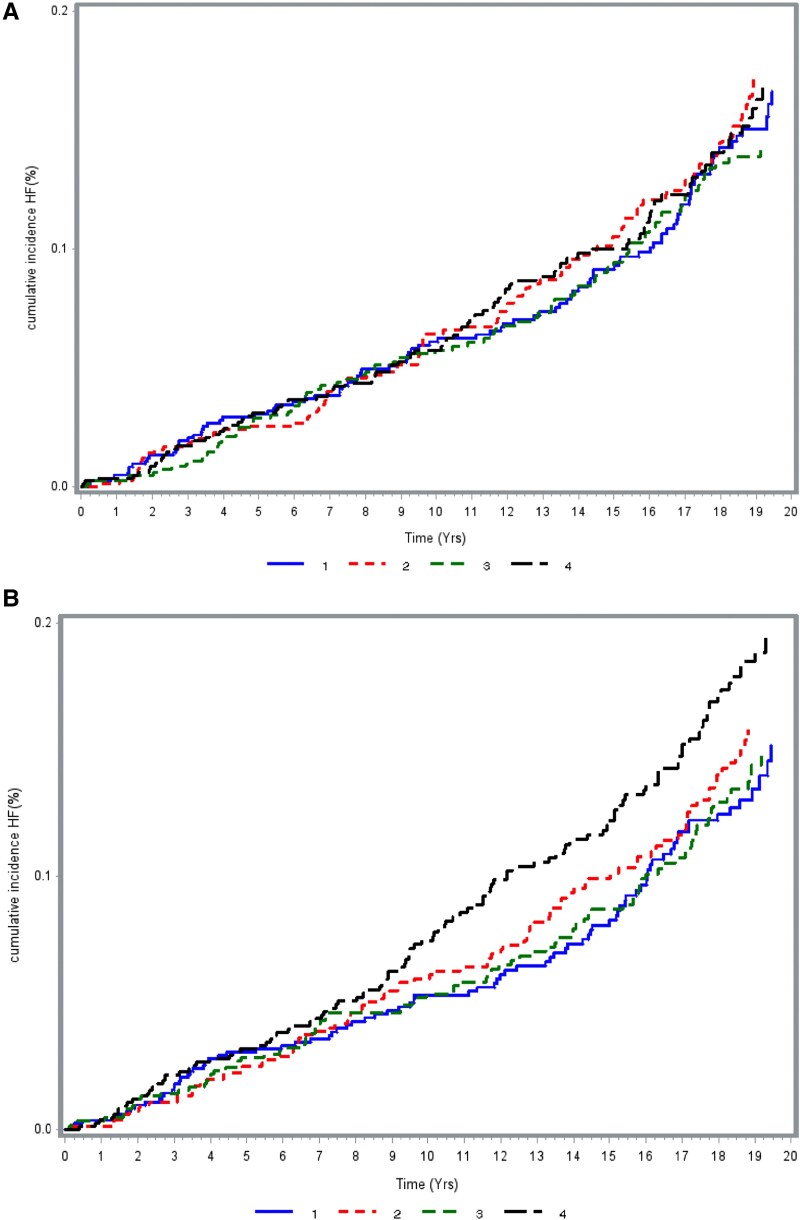
Cumulative heart failure incidence by quartiles of (*A*) total ketone body and (*B*) ketone body ratio

**Figure 2:**
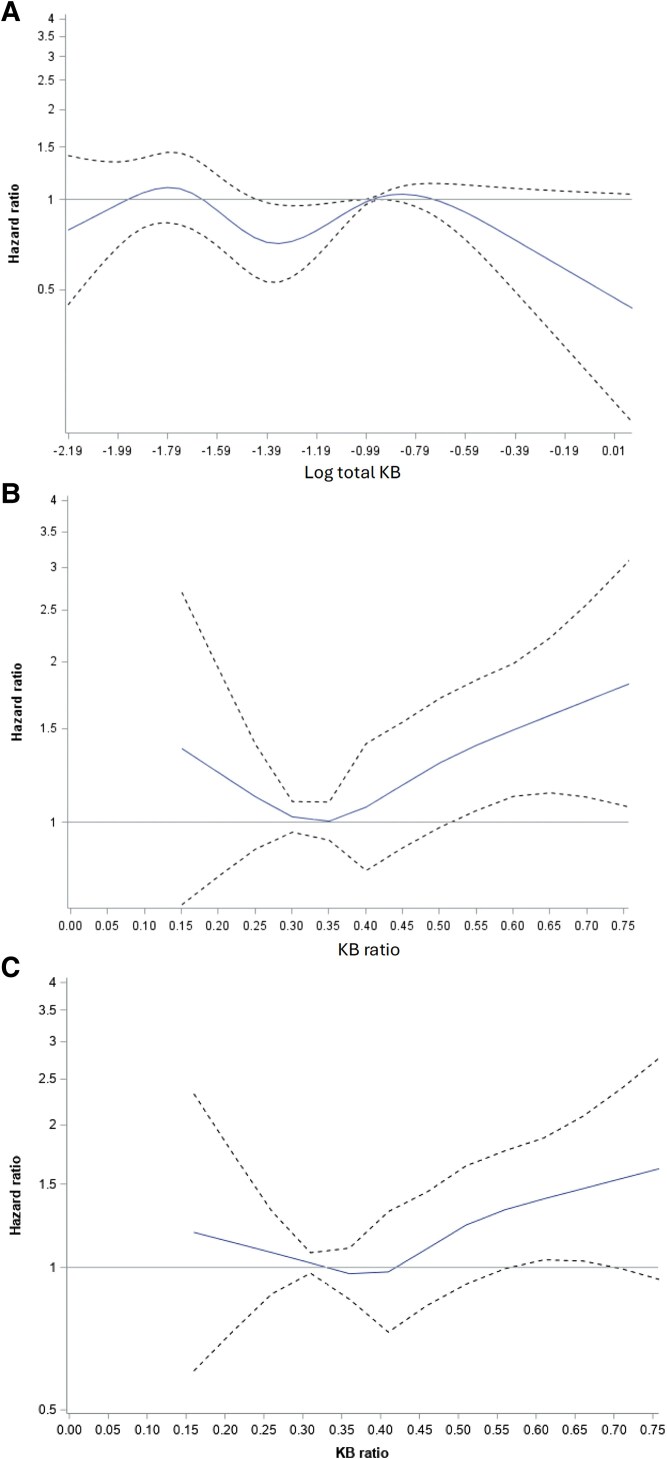
Association of age-adjusted risk of incident heart failure with (*A*) total ketone body and (*B*) ketone body ratio and (*C*) ketone body ratio with additional adjustment for N-terminal pro-B-type natriuretic peptide: total ketone and ketone body ratio modelled as restricted cubic splines with knots at the 5th, 25th, 50th, 75th, and 95th percentiles adjusted for age

The cumulative incidence curves are shown in *Figure 1A* and *B* for quartiles of total KB and quartiles of KB ratio respectively. The highest KB ratio quartile shows separation from other quartiles towards higher cumulative incidence that increases with follow-up time. Restricted cubic spline analysis, shown in *Figure 2*, modelled the relationship between (*Figure 2A*) total KB, (*Figure 2B*) KB ratio, and incidence of HF. While no clear association was demonstrated for total KB, higher KB ratio was associated with increased HF risk. Risk was slightly elevated at levels below 0.25 (bottom 5%). Importantly, from a KB ratio of 0.35 (33%) there appeared to be a linear increase in HF incidence hazard ratio with risk significantly raised at KB ratio above 0.55 (top decile). Further adjustment for NT-pro-BNP made minor differences to the pattern seen (*Figure 2C*).

*Table 4* shows the results of the Cox regression model analysing the association between incidence of HF with total KB and KB ratio by quartiles. Because of a possible threshold effect the top quartile of the KB ratio was further divided with the top decile separated, and five groups were used: Quartile 1 (0%–25% centile), Quartile 2 (25%–49% centile), Quartile 3 (50%–74% centile), Quartile 4 (75%–89% centile), and top decile. In the age-adjusted analysis, total KB was not significantly associated with incident HF [HR 0.94 (0.84–1.04), *P* = .231]. Similar to total KB findings, there was no association between β-hydroxybutyrate and acetoacetate with incident HF. Contrastingly, in the age-adjusted analysis, KB ratio was significantly associated with incident HF [HR 1.12 (1.01–1.24), *P* = .023] with risk significantly raised at KB ratio levels >0.55 (top decile). The association remained significant in the models adjusting for age, BMI, social class, systolic BP, BP medication, eGFR smoking, physical activity, total cholesterol, HDL, lipid medication, interleukin-6 and prevalent AF. Additionally adjusting for NT-pro-BNP made minor differences and those with levels >0.55 (top decile) showed significantly elevated risk.

**Table 4:** Association of total ketone body, β-hydroxybutyrate, acetoacetate, and ketone body ratio with incident heart failure

		Number of events	Incident HF			
				HR	95% CI	*P*-value
Total KB	Model 1	373	Standardized Log total KB	0.94	0.84–1.04	.231
			Q1	1.00		
			Q2	0.99	0.75–1.31	.922
			Q3	0.82	0.61–1.11	.198
			Q4	0.92	0.69–1.23	.566
β-OHB	Model 1	373	Standardized Log β-OHB	0.92	0.83–1.02	.112
			Q1	1.00		
			Q2	0.86	0.65–1.14	.307
			Q3	0.80	0.60–1.07	.126
			Q4	0.81	0.61–1.08	.159
Acetoacetate	Model 1	373	Standardized Log acetoacetate	0.98	0.88–1.08	.668
			Q1	1.00		
			Q2	0.92	0.69–1.22	.565
			Q3	0.81	0.61–1.09	.163
			Q4	0.97	0.73–1.28	.820
KB ratio	Model 1	373	Standardized KB ratio	1.12	1.01–1.24	.**023**
			Q1	1.00		
			Q2	0.97	0.70–1.33	.835
			Q3	1.10	0.82–1.46	.519
			Q4 (75–89%)	1.16	0.82–1.63	.396
			Top decile	1.66	1.19–2.31	.**003**
	Model 2	355	Standardized KB ratio	1.12	1.01–1.24	.**027**
			Q1	1.00		
			Q2	0.98	0.71–1.36	.867
			Q3	1.18	0.88–1.59	.364
			Q4 (75–89%)	1.18	0.83–1.67	.392
			Top decile	1.58	1.12–2.24	.**009**
	Model 3	341	Standardized KB ratio	1.11	1.00–1.23	.056
			Q1	1.00		
			Q2	1.00	0.71–1.39	.985
			Q3	1.09	0.80–1.47	.599
			Q4 (75–89%)	1.10	0.77–1.57	.610
			Top decile	1.60	1.13–2.27	.**008**

Adjusted relative hazard ratios and 95% confidence intervals (CIs) for incident heart failure, by quartiles of total KB, β-OHB, acetoacetate, and KB ratio, and with natural log transformed and standardized total KB, β-OHB, and acetoacetate values as well as KB ratio and standardized KB ratio values, fitted continuously, with no prevalent heart failure or myocardial infarction. Bold indicates *P*-value <.05. Model 1: Adjusted for age. Model 2: Model 1 + BMI, social class, systolic blood pressure, taking blood pressure medication, smoking, physical activity, estimated glomerular filtration rate, total cholesterol, HDL, taking lipid-lowering drugs, IL-6, and prevalent AF. Model 3: Model 2 + NT-pro-BNP. Significant p-values are indicated in bold.

When stratified by NT-pro-BNP levels, the ratio showed weak associations with HF in those with below the median NT-pro-BNP levels (NT-pro-BNP <83 pg/ml). However, among those with elevated NT-pro-BNP above the median, elevated KB ratio showed significantly raised risk compared to those with lower levels. The age-adjusted HR (95% CI) for the five groups were 1.00, 0.78 (0.44–1.4), 1.15 (0.71–1.81), 0.98 (0.54–1.80), and 1.23 (0.66–2.27) in those with below the median NT-pro-BNP levels and 1.00, 1.08 (0.72–1.61), 1.04 (0.72–1.50), 1.17 (0.76–1.80), and 1.87 (1.25–2.81) in those with levels above the median [NT-pro-BNP >83 (pg/ml)]. However, a formal test for interaction could not confirm a significant difference in the association between elevated KB ratio and NT-pro BNP with HF (*P* = .29) possibly because of small numbers. The increased risk was seen even after exclusion of those with NT-pro-BNP above 400 pg/ml (possible HF) (*n* = 41 men) [HR =1.77 (1.06–2.93)].

### Subgroup analysis

The association between KB ratio and incident HF was examined by age and diabetic status (Supplementary Table S3). KB ratio was significantly associated with incident HF in the <70 years age group but not the ≥70 age group. The age-adjusted HRs (95% CI) (KB ratio top decile vs lowest quartile) were 1.96 (1.27–3.01) and 1.33 (0.78–2.24) in men aged <70 and in men aged ≥70 respectively. The association between KB ratio and incident HF was more evident in those without diabetes. However, the number of men with diabetes was small. No associations were seen between total KB and incident HF in either subgroup.

### Association of plasma ketone bodies with HF subtype

We conducted a supplementary analysis examining the associations between KB and specific HF subtypes, restricting incident cases to those with information on ejection fraction (*n* = 214 cases). Total KB was not significantly associated with incident HFrEF or HFpEF. By contrast, KB ratio showed a significant association with HF subtype. These results are shown in Supplementary Table S4. Elevated KB ratio was associated with increased risk of both HFrEF and HFpEF incidence although the relationship between KB ratio and HFrEF was weaker and did not achieve statistical significance.

### Association of plasma ketone bodies with incident MI

Our results also confirmed no significant association between KB values and incident MI. Thus, in the age-adjusted cox model of KB quartiles neither total KB (Q4 vs Q1 HR 0.86; 95% CI 0.67 −1.09; *P* = .213) nor KB ratio (Q4 vs Q1 HR 0.87; 95% CI 0.68 −1.11; *P* = .261) were significantly associated with incident MI.

## Discussion

To our knowledge, this is the first epidemiological study that has investigated the association between KB ratio and incident HF finding that a higher KB ratio above 0.55 is associated with a significantly increased risk of incident HF. However, we observed no association between total KB or the individual KB (β-hydroxybutyrate and acetoacetate) with incident HF. Ketone body ratio represents a distinct biological parameter to total KB as evident by its differing relationships with demographic variables, lipid profile, inflammatory markers, and hepatic enzymes.

### KB ratio and incident HF

Risk of incident HF tended to increase with increasing KB ratio but was only significantly increased when the KB ratio was above 0.55 which represented 10% of the population study. Participants in the top decile of KB ratio had a 66% increased risk of incident of HF versus the lowest quartile. This association remained significant after adjustment for key cardiovascular risk factors including age, BMI, social class, systolic BP, taking BP medication, smoking, alcohol, physical activity, eGFR, total cholesterol, HDL, taking lipid-lowering drugs, prevalent AF, and interleukin-6. Additional adjustment for NT-pro-BNP made minor differences. However, elevated KB ratio appeared to interact with NT-pro-BNP, and HF risk was only increased when NT-pro-BNP was also raised. Natriuretic peptides have been shown to stimulate lipolysis resulting in increased KB levels.^20,21^ Thus, increased KB ratio levels associated with raised NT-pro-BNP appear to be linked to HF risk.

Ketone body ratio, defined as the ratio of acetoacetate to β-hydroxybutyrate, reflects mitochondrial redox potential (NAD^+^/NADH) whereby acetoacetate and β-hydroxybutyrate are in equilibrium with NAD^+^/NADH ratio (high NADH) driven by β-hydroxybutyrate dehydrogenase in the mitochondria.^2,22^ NAD^+^ serves as a cofactor in fuel breakdown, whereas NADH is a substrate for complex I in the electron transport chain facilitating oxidative phosphorylation and the antioxidant reactive oxygen species scavenging system via nicotinamide nucleotide transhydrogenase.^23^ Therefore, the NAD^+^/NADH ratio reflects the overall status of mitochondrial metabolism. Under basal conditions when metabolic substrates are plentiful and ATP demand minimal, NADH levels are high and the NAD^+^/NADH ratio, and hence KB ratio, is low.

Several biological mechanisms may underlie the observed association between higher KB ratio and incident HF. First, an elevated KB ratio may reflect neurohormonal activation along the hepato-cardiac axis, in which natriuretic peptide–driven lipolysis increases hepatic free fatty acid flux and ketogenesis.^24^ This process raises circulating acetoacetate relative to β-hydroxybutyrate and could therefore elevate KB ratio as an early marker of sub-clinical myocardial stress. The finding that elevated KB ratio was associated with HF risk only in those with elevated NT-pro-BNP as well would be supportive of this pathway. Second, because the acetoacetate–β-hydroxybutyrate pair is in equilibrium with the mitochondrial NAD⁺/NADH redox couple, a higher KB ratio may signal redox imbalance.^25^ Chronic redox imbalance can impair mitochondrial oxidative phosphorylation, promote oxidative stress, and contribute to myocardial remodelling.^26^ Third, failing hearts undergo profound metabolic remodelling with a shift in substrate utilization including greater reliance on KB.^4^ Thus, a rise in KB ratio may represent an adaptive metabolic response to early myocardial energetic stress that occurs in the early phases of HF syndrome development. An elevated KB ratio may therefore signal mitochondrial redox imbalance and serve as a biomarker of HF risk. Further biochemical investigations are necessary to provide mechanistic explanations to these epidemiological findings. Elucidating these pathways may offer important novel therapeutic strategies in HF.

### Comparison with previous prospective studies exploring the association between KB and incident HF

*Table 5* summarizes the key results and relevant demographic information of the epidemiological prospective cohort studies which investigated the association between KB and incident HF. To date, eight prospective studies have examined the association between total KB or its individual components (β-hydroxybutyrate and acetoacetate) with incident HF.

**Table 5:** Summary of previous prospective studies exploring the association between ketone bodies and incident heart failure

Study	Cases/total	Age (mean)	Sex (% male)	Follow-up (years)	Country	HF type	Ketone body	Key findings
MESA^8^	385/6796	62	47	15.7	USA	Not specified	Total KB	Significantly associated with increased risk of HF (HR 1.68, CI 1.07–2.65, *P* = .025, adjusted for age, sex, race/ethnicity, education level, BMI, diabetes, smoking, systolic BP, BP medications, total cholesterol, HDL cholesterol, lipid-lowering therapy, eGFR, physical activity, total calorie intake, and NT-pro-BNP).
								β-Hydroxybutyrate and acetoacetate showed similar results.
MESA^27^	Same cohort as above							Found that the addition of total KB to NT-pro-BNP and high sensitive cardiac troponin T (hs-cTnT) improved 5- and 10-year incident HF risk prediction (C-statistic 0.74 vs 0.77, *P* = .02 and 0.70 vs 0.73, *P* = .02, respectively).
UK Biobank^28^	3150/117 498	58	46	10	UK	Not specified	β-hydroxybutyrate	Only weak magnitude of association with HF. Specific numerical values for the HR and CI were not provided, visual estimate of HR ∼1.1 though unclear if reached the study wide significance threshold. Adjusted for age, sex, and assessment centre.
CHS^11^	637/1758	72.7	40.7	20	USA	Both types	Total KB	Not statistically significantly associated with increased risk of HF (HR 1.2, CI 1.0–1.4, *P* = .08, adjusted for age, sex, clinic site, ethnicity, education level, kcals physical activity per week, BMI, BMI2, smoking status, and alcoholic drinks per week, fasting >8 hours, hypertension, cholesterol, triglycerides, diagnosis of diabetes, eGFR, hypertension medication use, lipid-lowering medication use, and oestrogen use). The model adjusting for fewer confounders (age, sex, clinic site, ethnicity, education level, kcals physical activity per week, BMI, BMI2, smoking status, and alcoholic drinks per week, fasting >8 h) was statistically significantly associated with increased risk of HF (HR 1.2, CI 1.0–1.3). However, when total KB was analysed as quintiles rather than continuous variables, none of the models were statistically significant.
								In the supplementary analysis, total KB was statistically significant in: Younger (<median age) but not older participants. Those with <median BMI but not >median BMI. Those who develop HFrEF but not HFpEF.
CANVAS^12^	136/3581	62.7	64.2	6.1	667 centres in 30 countries	Not specified	β-hydroxybutyrate	Not significantly associated with incident HF (HR 0.93, CI 0.65–1.36, adjusted for sex, age, BMI, eGFR, HbA1c, systolic BP, prior cardiovascular disease (CVD), prior HF, smoking, urine albumin-to-creatinine ratio (UACR), HDL cholesterol, LDL cholesterol, and use of statins, renin-angiotensin-aldosterone-system (RAAS) inhibitors, antithrombotics, and loop diuretics, in addition to seven log-transformed substrates).
							Acetoacetate	Not significantly associated with incident HF (HR 1.06, CI 0.66–1.71).
METISM^9^	172/10 106	58	100	8.8	Finland (Eastern Finland)	Both types (52.9% HFpEF)	Acetoacetate	Significantly associated with increased incident HF (HR 1.14, CI 1.05–1.24, *P* = .027, adjusted age, BMI, diabetes, and statin medication).
							β-hydroxybutyrate	Not significantly associated with incident HF.
PREVEND^13^	227/6134	53.3	49.3 men	8.2	Netherlands	Both types (but later provide subgroup analysis)	β-hydroxybutyrate	Not significantly associated with incident HF (HR 1.18, CI 0.99–1.41, *P* = .05, adjusted for age + sex + BMI + T2D + hypertension + MI + smoking + alcohol consumption + total cholesterol + HDL-C + triglycerides + leucine + glucose + eGFR + urinary albumin excretion ( UAE). Further adjusted model similarly not significant, whereas less adjusted models were significant.
								In the subgroup analysis, β-hydroxybutyrate was significantly associated with incident HF in female but not male participants, and with HFrEF but not HFpEF.
PROSPER^10^	182/5341	75.8	48.5	2.7	Scotland, Ireland, Netherlands	Not specified (but HF definition biased towards inclusion HFrEF)	β-hydroxybutyrate	Significantly associated with increased incident HF (HR 1.19, CI 1.025–1.38, *P* = .021, adjusted for sex, BMI, SBP, diastolic blood pressure (DBP), current smoking, diabetes, pravastatin/placebo, BP-lowering therapy, major coronary events/baseline cardiovascular disease). However, was not statistically significant in the model accounting for NT-pro-BNP.
FINRISK^10^	133/7330	48.8	50.8	5	Finland	Not specified	β-hydroxybutyrate	Significantly associated with decreased incident of HF (HR 0.811, CI 0.663–0.992, *P* = .041, adjusted for sex, SBP, DBP, smoking, diabetes, lipid-lowering therapy, BP-lowering therapy, prevalent CVD, eGFR, and NT-pro-BNP).
							Acetoacetate	Not significantly associated with incident HF (HR 0.933, CI 0.785–1.11, *P* = .443).

Prior observational studies have reported inconsistent associations between KB and incident HF (*Table 5*)—five showing positive associations and three reporting null findings. These discrepancies likely stem from differences in study methodologies and populations highlighted by the significantly different rate of HF events.

Firstly, age appears to influence the relationship between KB and incident HF. In our study, KB ratio showed a stronger association with HF among participants <70 years whereby in the age-adjusted model the highest KB ratio decile had a 96% increased risk of incident HF versus the lowest quartile compared to a 33% increase in the older men (≥70 years). The Cardiovascular Health Study reported a similar age-dependent effect on their association between total KB and incident HF.^11^

Secondly, sex is also likely an important confounder. Our results only involve male participants and thus do not allow comparison based on sex. Nevertheless, the PREVEND study demonstrated that the relationship between β-hydroxybutyrate and incident HF was significant for female participants only.^13^ This may explain our null findings for total KB.

Thirdly, ethnicity also varied across studies. The BRHS, a UK-based study, involved almost exclusively (>99%) White participants. This contrasts with the US-based MESA study which included White (38.5%), Black (27.7%), Hispanic (22.0%), and Chinese American (11.8%) participants.^8^

Fourthly, study design differences including different follow-up methods, HF definitions, and inclusion criteria (e.g. prior MI), also complicate comparisons. For instance, the METSIM study found that including participants with previous MI altered the association between acetoacetate and HF,^9^ while both our study and MESA excluded such individuals.

Taken together, these comparisons suggest that heterogeneity in age, sex, and ethnicity across cohorts, as well as differences in study design and inclusion criteria, largely explain the conflicting findings in the literature. By investigating KB ratio in a well-characterized cohort, free of prior MI, and with long-term prospective follow-up, our study extends previous work through its robust methodology and identifies a novel biomarker that appears to precede the development of clinical HF.

### Subtype of HF

HF subtype, HFrEF and HFpEF, differ in their pathophysiology and patient profiles. HFpEF patients are typically older and more often females. HFpEF is often preceded by chronic comorbidities such as hypertension and obesity, whereas HFrEF is often preceded by ischaemia, myocarditis, or valvular disease.^29^ In our results, total KB was not significantly associated with the incidence of either HFrEF or HFpEF. In contrast, higher KB ratio was associated with increased risk of both HFrEF and HFpEF but was more strongly seen for HFpEF. However, the number of HFpEF cases was small and we did not have complete information on HF types in all men to make strong inference about types of HF.

Only two prior epidemiological studies have investigated HF subtype in relation to KB. The PREVEND study found that higher β-hydroxybutyrate was associated with an increased risk of HFrEF but not with HFpEF.^13^ Similarly, the Cardiovascular Health Study found that total KB was significantly associated with increased risk of HFrEF but not HFpEF incidence.^11^

### Limitations

The BRHS is a large prospective cohort study, reporting findings from multiple detailed assessments, with long follow-up times. In the current study we provide important novel findings relating to KB ratio and incident HF as well as shed light into the discrepancies of previous findings regarding the relationship between total KB and incident HF. However, there are limitations to this work. These results describe epidemiological relationships and are subject to limitations affecting all observational research including an inability to establish causality. HF incidence is based on physician-diagnosed HF, which likely underestimates true HF incidence in the population. Follow-up echocardiographic data were also incomplete, meaning not all participants could be confidently classified into HFrEF or HFpEF. Finally, the study population was older, predominantly white, entirely male, and free of prior MI, which limits the generalizability of the present results to women, other ethnic groups, and those with prior ischaemic heart disease. Nevertheless, as discussed previously, the ability to analyse the relationship between KB and incident HF in a specific uniform population can be considered a strength of the study and helped elucidate the discrepancies complicating the findings in the wider literature.

### Clinical implications and practical perspectives

International HF guidelines, such as the AHA/ACC/HFSA HF Guideline,^30^ endorse the use of validated multivariable risk scores to identify individuals at increased risk for developing HF. These scores typically integrate clinical parameters and serum biomarkers to enhance risk stratification. This is the first prospective study to examine and report an association between KB ratio and incident HF independent of traditional cardiovascular risk factors and inflammatory markers. Therefore, KB ratio may serve as a novel biomarker for identifying individuals with no prior MI who are at an increased risk of developing HF, particularly when NT-pro-BNP levels are elevated. Future studies should aim to validate these findings in more diverse populations, including women and other ethnic groups, to determine the generalizability of the KB ratio as a risk marker and to clarify its value within multivariable prediction models. Incorporating KB ratio into existing risk prediction models for HF could potentially improve their performance by capturing a distinct metabolic dimension of HF susceptibility.

Additionally, KB ratio may provide insights into the metabolic pathways that contribute to HF pathogenesis. As a reflection of mitochondrial redox balance, it may implicate early metabolic dysfunction predisposing to HF or compensatory mechanisms aimed at preserving cardiac energetics. Elucidating these pathways through dedicated mechanistic and experimental studies may ultimately inform the development of novel pharmacological strategies for HF prevention and treatment.

## Conclusions

This study reports important novel findings strongly associating KB ratio with incident HF in older British men. This association was more prominent in younger participants or in those with elevated NT-pro-BNP. There was no evidence that this relationship is influenced by diabetic status. In contrast to some previous studies, we found no association between total KB and incident HF. Careful examination of previous results suggests that the relationship between total KB and incident HF is likely limited to young female participants who develop HFrEF.

## Supplementary Material

xvag015_Supplementary_Data

## Declarations

### Data Availability

The BRHS data supporting this study are available from UKDS at 10.5255/UKDA-SN-9402-1 under controlled data access.

### Pre-registered Clinical Trial Number

None supplied.
