# Supplementary material for: Ketone bodies and incident heart failure: 20-year results from the prospective British Regional Heart Study
Source: ESC Heart Fail. 2026 Jan 14;13(1):xvag015. doi: 10.1093/eschf/xvag015 (PMC13108305; doi:10.1093/eschf/xvag015)
Supplement: xvag015_Supplementary_Data [file xvag015_supplementary_data.docx]

**Supplementary Tables:**

**Supplementary table 1: Baseline characteristics and biochemical profile by β-hydroxybutyrate** **quartiles**

|  | **Q1 (<0.1300**  **mmol/L) (*n* = 849)** | **Q2 (0.1300 - 0.1750**  **mmol/L) (*n* = 868)** | **Q3 (0.1750 - 0.2650**  **mmol/L) (*n* = 870)** | **Q4 (>0.2650 mmol/L) (*n* = 862)** | ***P* value** |
| --- | --- | --- | --- | --- | --- |
| **Age (years)** | 67.6 (5.32) | 68.18 (5.54) | 68.96 (5.55) | 69.22 (5.36) | **<0.0001** |
| **BMI (kg/m^2^)** | 27.04 (3.62) | 27.14 (3.64) | 26.78 (3.54) | 26.27 (3.45) | **<0.0001** |
| **Current smoker (%)** | 117 (13.8) | 111 (12.8) | 110 (12.67) | 106 (12.31) | 0.139 |
| **Physical activity (% inactive or only occasionally active)** | 257 (30.27) | 296 (34.1) | 258 (29.66) | 280 (32.48) | 0.160 |
| **Heavy alcohol use (%)** | 19 (2.26) | 16 (1.87) | 23 (2.69) | 35 (4.12) | **0.027** |
| **Social class (% manual occupation)** | 413 (48.7) | 453 (52.25) | 446 (51.38) | 435 (50.7) | 0.764 |
| **Systolic BP (mmHg)** | 147.85 (24.55) | 148.92 (23.02) | 151.06 (23.62) | 152.17 (23.68) | **0.0005** |
| **Diastolic BP (mmHg)** | 84.49 (10.89) | 85.54 (10.8) | 85.43 (10.74) | 87.08 (11.28) | **<0.0001** |
| **Hypertension diagnosis (%)** | 258 (31.81) | 250 (29.94) | 236 (28.82) | 213 (25.76) | 0.0527 |
| **Taking BP-lowering medication (%)** | 265 (31.93) | 241 (27.96) | 218 (25.41) | 212 (24.91) | **0.005** |
| **Total cholesterol (mmol/L)** | 5.87 (1.02) | 6.07 (1.07) | 6.14 (1.05) | 6.11 (1.08) | **<0.0001** |
| **LDL (mmol/L)** | 3.73 (0.92) | 3.92 (0.94) | 4 (0.96) | 4 (0.96) | **<0.0001** |
| **HDL (mmol/L)** | 1.29 (0.31) | 1.27 (0.32) | 1.35 (0.34) | 1.4 (0.36) | **<0.0001** |
| **Triglycerides** **(mmol/L)** | 1.72 (1.18 - 2.41) | 1.79 (1.27 - 2.38) | 1.62 (1.18 - 2.13) | 1.40 (1.02 - 1.88) | **<0.0001** |
| **Taking lipid-lowering medication (%)** | 40 (4.71) | 38 (4.38) | 40 (4.6) | 33 (3.83) | 0.814 |
| **DM (%)** | 143 (16.84) | 116 (13.36) | 62 (7.13) | 59 (6.84) | **<0.0001** |
| **Insulin (pmol/L)** | 9.58 (6.14 - 13.48) | 9.21 (5.96 - 12.88) | 7.92 (5.55 - 11.27) | 7.39 (5.08 - 10.66) | **<0.0001** |
| **NT-proBNP (pg/ml)** | 79.834 (37 - 165) | 82.27 (41 - 155) | 89.12 (45 - 163.5) | 103.54 (51 - 188) | **<0.0001** |
| **Troponin T (pg/ml)** | 14.28 (44.98) | 12.97 (8.23) | 13.23 (7.6) | 13.74 (9.32) | 0.669 |
| **CRP (mg/L)** | 1.48 (0.69 - 2.97) | 1.73 (0.82 - 3.46) | 1.75 (0.85 - 3.41) | 1.77 (0.81 - 3.64) | **0.002** |
| **IL-6 (pg/ml)** | 2.14 (1.38 - 2.98) | 2.32 (1.49 - 3.32) | 2.51 (1.64 - 3.4) | 2.64 (1.68 - 3.79) | **<0.0001** |
| **Estimated GFR (ml/min/1.73 m^2^)** | 73.51 (11.77) | 72.81 (11.92) | 72.62 (13.98) | 72.38 (12.77) | 0.293 |
| **GGT** **(u/l)** | 28.22 (19 - 39) | 28.79 (20 - 40) | 28.50 (19 - 38) | 28.22 (18 - 37) | 0.864 |
| **ALT (u/l)** | 16.12 (13 - 21) | 16.44 (12 - 21) | 15.49 (12 - 20) | 15.03 (12 - 19) | **<0.001** |
| **Albumin g/L** | 44.01 (2.62) | 44.16 (2.74) | 44.35 (2.68) | 44.25 (2.79) | 0.060 |
| **Atrial fibrillation (%)** | 25 (2.95) | 27 (3.12) | 21 (2.42) | 35 (4.07) | 0.264 |
| **Incident HF (%)** | 102 (12.01) | 94 (10.83) | 89 (10.23) | 88 (10.21) | 0.593 |
| **Incident MI (%)** | 126 (14.84) | 134 (15.44) | 115 (13.22) | 128 (14.85) | 0.593 |
| **Incident stroke (%)** | 92 (10.84) | 105 (12.1) | 108 (12.41) | 107 (12.41) | 0.712 |

*For normally distributed continuous variables, values are mean (standard deviation). For insulin, CRP, IL-6, total triglycerides, GGT, ALT, adiponectin, and NT-pro-BNP values are geometric mean (inter-quartile range). p values for continuous variables were calculated with ANOVA. For categorical variables values are N (% of total) and p values for χ2 tests.*

**Supplementary table 2: Baseline characteristics and biochemical profile by acetoacetate** **quartiles**

|  | **Q1 (<0.0483**  **mmol/L) (*n* = 864)** | **Q2 (0.0483 - 0.072**  **mmol/L) (*n* = 867)** | **Q3 (0.072 - 0.117**  **mmol/L) (*n* = 872)** | **Q4 (>0.117 mmol/L) (*n* = 856)** | ***P* value** |
| --- | --- | --- | --- | --- | --- |
| **Age (years)** | 67.57 (5.43) | 68.58 (5.43) | 68.81 (5.57) | 69.01 (5.38) | **<0.0001** |
| **BMI (kg/m^2^)** | 26.88 (3.66) | 27.02 (3.48) | 27.01 (3.72) | 26.28 (3.39) | **<0.0001** |
| **Current smoker (%)** | 126 (14.62) | 104 (12) | 105 (12.06) | 111 (13) | 0.090 |
| **Physical activity (% inactive or only occasionally active)** | 272 (31.48) | 297 (34.26) | 279 (32) | 247 (28.86) | 0.380 |
| **Heavy alcohol use (%)** | 21 (2.46) | 17 (2) | 25 (2.91) | 36 (4.26) | **0.036** |
| **Social class (% manual occupation)** | 451 (52.26) | 447 (51.68) | 449 (51.61) | 407 (47.71) | 0.195 |
| **Systolic BP (mmHg)** | 148.37 (23.96) | 150.6 (24.53) | 150.04 (22.84) | 151.13 (23.8) | 0.090 |
| **Diastolic BP (mmHg)** | 84.83 (10.6) | 85.68 (11.13) | 85.56 (10.94) | 86.53 (11.2) | **0.016** |
| **Hypertension diagnosis (%)** | 255 (30.72) | 259 (31.39) | 229 (27.79) | 217 (26.4) | 0.080 |
| **Taking BP-lowering medication (%)** | 239 (28.12) | 252 (29.58) | 226 (26.22) | 220 (25.97) | 0.295 |
| **Total cholesterol (mmol/L)** | 5.99 (1.05) | 6.03 (1.07) | 6.06 (1.05) | 6.1 (1.07) | 0.169 |
| **LDL (mmol/L)** | 3.87 (0.94) | 3.89 (0.97) | 3.91 (0.94) | 3.99 (0.97) | 0.053 |
| **HDL (mmol/L)** | 1.32 (0.31) | 1.29 (0.33) | 1.33 (0.34) | 1.39 (0.37) | **<0.0001** |
| **Triglycerides** **(mmol/L)** | 1.63 (1.16 - 2.18) | 1.77 (1.26 - 2.43) | 1.67 (1.19 - 2.22) | 1.45 (1.03 - 1.95) | **<0.0001** |
| **Taking lipid-lowering medication (%)** | 35 (4.05) | 32 (3.69) | 40 (4.59) | 44 (5.14) | 0.478 |
| **DM (%)** | 132 (15.28) | 116 (13.38) | 84 (9.63) | 48 (5.61) | **<0.0001** |
| **Insulin (pmol/L)** | 9.30 (5.8 - 12.9) | 9.03 (5.94 - 12.95) | 8.41 (5.8 - 12.01) | 7.32 (5.09 - 10.48) | **<0.0001** |
| **NT-proBNP (pg/ml)** | 75.94 (37 - 155) | 87.36 (44 - 160) | 90.92 (45 - 169) | 100.48 (48 - 191) | **<0.0001** |
| **Troponin T (pg/ml)** | 12.78 (7.9) | 14.45 (44.42) | 13.42 (8.66) | 13.57 (9.15) | 0.530 |
| **CRP (mg/L)** | 1.54 (0.74 - 3.1) | 1.68 (0.8 - 3.26) | 1.73 (0.85 - 3.57) | 1.77 (0.82 - 3.5) | 0.058 |
| **IL-6 (pg/ml)** | 2.20 (1.4 - 3.1) | 2.27 (1.51 - 3.29) | 2.51 (1.61 - 3.46) | 2.64 (1.7 - 3.68) | **<0.0001** |
| **Estimated GFR (ml/min/1.73 m^2^)** | 73.38 (11.92) | 72.39 (11.93) | 72.86 (14.11) | 72.73 (12.54) | 0.438 |
| **GGT** **(u/l)** | 28.50 (18 - 40) | 28.50 (19 - 38) | 27.94 (19 - 38) | 28.79 (19 - 39) | 0.745 |
| **ALT (u/l)** | 16.28 (12 - 21) | 15.64 (12 - 20) | 15.64 (12 - 20) | 15.49 (12 - 20) | 0.045 |
| **Albumin g/L** | 44.18 (2.68) | 44.11 (2.7) | 44.21 (2.8) | 44.26 (2.65) | 0.712 |
| **Atrial fibrillation (%)** | 16 (1.86) | 30 (3.46) | 23 (2.64) | 39 (4.57) | **0.009** |
| **Incident HF (%)** | 97 (11.23) | 96 (11.07) | 85 (9.75) | 97 (11.33) | 0.689 |
| **Incident MI (%)** | 136 (15.74) | 128 (14.76) | 108 (12.39) | 132 (15.42) | 0.186 |
| **Incident stroke (%)** | 94 (10.88) | 121 (13.96) | 86 (9.86) | 112 (13.08) | **0.030** |

*For normally distributed continuous variables, values are mean (standard deviation). For insulin, CRP, IL-6, total triglycerides, GGT, ALT, adiponectin, and NT-pro-BNP values are geometric mean (inter-quartile range). p values for continuous variables were calculated with ANOVA. For categorical variables values are N (% of total) and p values for χ2 tests.*

**Supplementary Table 3. Association of ketone body ratio with incident heart failure in men <70 years and those ≥70 years, and in diabetic and non-diabetic men.**

| **Association of ketone body ratio with incident heart failure in men <70 years to those ≥70 years** | | | | | | | | | | |
| --- | --- | --- | --- | --- | --- | --- | --- | --- | --- | --- |
|  | | **Age < 70 years** | | | | **Age ≥ 70 years** | | | | |
|  |  | **Number of events** | **HR** | **95% CI** | **P value** | **Number of events** | **HR** | **95% CI** | **P value** |  |
| **Model 1** |  | 201 |  |  |  | 172 |  |  |  |  |
|  | Standardised KB ratio |  | 1.16 | 1.02 - 1.33 | **0.023** |  | 1.07 | 0.92 - 1.25 | 0.371 |  |
|  | Q1 |  | 1.00 |  |  |  | 1.00 |  |  |  |
|  | Q2 |  | 1.11 | 0.73-1.70 | 0.618 |  | 0.81 | 0.50-1.33 | 0.409 |  |
|  | Q3 |  | 1.03 | 0.69-1.53 | 0.891 |  | 1.16 | 0.77-1.77 | 0.477 |  |
|  | Q4 (75-89%) |  | 1.21 | 0.76-1.95 | 0.419 |  | 1.10 | 0.67-1.80 | 0.702 |  |
|  | Top decile |  | 1.96 | 1.27-3.01 | **0.002** |  | 1.33 | 0.78-2.24 | 0.290 |  |
| **Model 2** |  | 195 |  |  |  | 160 |  |  |  |  |
|  | Standardised KB ratio |  | 1.17 | 1.02 - 1.34 | **0.024** |  | 1.05 | 0.90 - 1.24 | 0.510 |  |
|  | Q1 |  | 1.00 |  |  |  | 1.00 |  |  |  |
|  | Q2 |  | 1.13 | 0.73-1.75 | 0.513 |  | 0.79 | 0.47-1.31 | 0.361 |  |
|  | Q3 |  | 1.14 | 0.76-1.72 | 0.538 |  | 1.20 | 0.77-1.85 | 0.423 |  |
|  | Q4 (75-89%) |  | 1.29 | 0.79-2.08 | 0.306 |  | 1.03 | 0.62-1.72 | 0.903 |  |
|  | Top decile |  | 2.00 | 1.27-3.13 | **0.003** |  | 1.11 | 0.63-1.94 | 0.720 |  |
| **Association of ketone body ratio with incident heart failure in diabetic and non-diabetic men** | | | | | | | | | |  |
|  | | **Non-diabetic** | | | | **Diabetic** | | | |  |
|  |  | **Number of events** | **HR** | **95% CI** | **P value** | **Number of events** | **HR** | **95% CI** | **P value** |  |
| **Model 1** |  | 324 |  |  |  | 49 |  |  |  |  |
|  | Standardised KB ratio |  | 1.12 | 1.00 - 1.25 | **0.042** |  | 1.13 | 0.88 -1.45 | 0.338 |  |
|  | Q1 |  | 1.00 |  |  |  | 1.00 |  |  |  |
|  | Q2 |  | 0.94 | 0.66-1.33 | 0.721 |  | 1.23 | 0.56-2.72 | 0.603 |  |
|  | Q3 |  | 1.12 | 0.82-1.52 | 0.471 |  | 1.14 | 0.51-2.57 | 0.751 |  |
|  | Q4 (75-89%) |  | 1.22 | 0.85-1.76 | 0.202 |  | 0.84 | 0.30-2.32 | 0.731 |  |
|  | Top decile |  | 1.71 | 1.19-2.49 | **0.004** |  | 1.39 | 0.58-3.32 | 0.456 |  |
|  |  |  |  |  |  |  |  |  |  |  |
| **Model 2** |  | 308 |  |  |  | 47 |  |  |  |  |
|  | Standardised KB ratio |  | 1.12 | 1.00 -1.26 | **0.045** |  | 1.04 | 0.80 - 1.36 | 0.750 |  |
|  | Q1 |  | 1.00 |  |  |  | 1.00 |  |  |  |
|  | Q2 |  | 0.97 | 0.68-1.39 | 0.862 |  | 1.26 | 0.54-2.94 | 0.588 |  |
|  | Q3 |  | 1.21 | 0.88-1.66 | 0.249 |  | 1.29 | 0.54-3.08 | 0.566 |  |
|  | Q4 (75-89%) |  | 1.24 | 0.86-1.81 | 0.254 |  | 0.69 | 0.23-2.06 | 0.504 |  |
|  | Top decile |  | 1.64 | 1.13-2.39 | **0.010** |  | 1.12 | 0.44-2.87 | 0.810 |  |

*Adjusted relative hazard ratios and 95% confidence intervals (CI) for incident heart failure, by quartiles of ketone body ratio with the top decile separated and standardised ketone body ratio values, fitted continuously, with no prevalent heart failure or myocardial infarction. Bold indicates p-value < 0.05.*

*Model 1: Adjusted for age*

*Model 2: Model 1 + BMI, social class, systolic blood pressure, taking blood pressure medication, smoking, alcohol, physical activity, estimated glomerular filtration rate, total cholesterol, HDL, taking lipid-lowering drugs, prevalent atrial fibrillation, and interleukin-6.*

**Supplementary Table 4.** Association of ketone body ratio with incident heart failure with reduced ejection fraction (HFrEF) and heart failure with preserved ejection fraction (HFpEF).

|  | | **HFrEF** | | | | **HFpEF** | | | | |
| --- | --- | --- | --- | --- | --- | --- | --- | --- | --- | --- |
|  |  | **Number of events** | **HR** | **95% CI** | **P value** | **Number of events** | **HR** | **95% CI** | **P value** |  |
| **Model 1** |  | 155 |  |  |  | 59 |  |  |  |  |
|  | Standardised KB ratio |  | 1.06 | 0.90-1.24 | 0.472 |  | 1.32 | 1.04-1.68 | 0.022 |  |
|  | Q1 |  | 1.00 |  |  |  | 1.00 |  |  |  |
|  | Q2 |  | 0.96 | 0.59-1.57 | 0.866 |  | 1.13 | 0.46-2.70 | 0.798 |  |
|  | Q3 |  | 1.10 | 0.71-1.71 | 0.665 |  | 1.58 | 0.73-3.46 | 0.249 |  |
|  | Q4 (75-89%) |  | 1.26 | 0.76-2.10 | 0.368 |  | 2.38 | 1.04-5.43 | 0.040 |  |
|  | Top decile |  | 1.47 | 0.87-2.50 | 0.158 |  | 2.49 | 1.04-5.98 | **0.042** |  |
|  |  |  |  |  |  |  |  |  |  |  |
| **Model 2** |  | 154 |  |  |  | 58 |  |  |  |  |
|  | Standardised KB ratio |  | 1.05 | 0.90-1.24 | 0.423 |  | 1.34 | 1.06-1.70 | 0.015 |  |
|  | Q1 |  | 1.00 |  |  |  | 1.00 |  |  |  |
|  | Q2 |  | 0.97 | 0.59-1.58 | 0.890 |  | 1.15 | 0.45-2.94 | 0.763 |  |
|  | Q3 |  | 1.16 | 0.74-1.80 | 0.524 |  | 1.86 | 0.83-4.20 | 0.134 |  |
|  | Q4 (75-89%) |  | 1.32 | 0.79-2.21 | 0.286 |  | 2.56 | 1.00-6.03 | 0.032 |  |
|  | Top decile |  | 1.44 | 0.84-2.48 | 0.184 |  | 2.63 | 1.06-6.56 | **0.038** |  |

*Adjusted relative hazard ratios and 95% confidence intervals (CI) for incident heart failure, by quartiles of ketone body ratio with the top decile separated, with ketone body ratio and standardised ketone body ratio values, fitted continuously, with no prevalent heart failure or myocardial infarction. Bold indicates p-value < 0.05.*

*Model 1: Adjusted for age*

*Model 2: Model 1 + BMI, social class, systolic blood pressure, taking blood pressure medication, smoking, physical activity, total cholesterol, HDL, taking lipid-lowering drug, IL-6 and atrial fibrillation.*
